# Cortical and spinal responses to short-term strength training and detraining in young and older adults in rectus femoris muscle

**DOI:** 10.1007/s00421-024-05443-0

**Published:** 2024-03-05

**Authors:** Gonzalo Gomez-Guerrero, Janne Avela, Ilkka Jussila, Esa Pihlajamäki, Fu-Yu Deng, Dawson J. Kidgell, Juha P. Ahtiainen, Simon Walker

**Affiliations:** 1 Faculty of Sport and Health Sciences, NeuroMuscular Research Center, Viveca, VIV221, University of Jyväskylä, 40700 Jyväskylä, Finland; 2 Monash Exercise Neuroplasticity Research Unit, School of Primary and Allied Health Care, Faculty of Medicine, Nursing and Health Sciences, Monash University, Melbourne, Australia

**Keywords:** Aging, resistance training, TMS, Lumbar stimulation, Cortico-spinal excitability, Lower-limbs

## Abstract

**Introduction:**

Strength training mitigates the age-related decline in strength and muscle activation but limited evidence exists on specific motor pathway adaptations.

**Methods:**

Eleven young (22–34 years) and ten older (66–80 years) adults underwent five testing sessions where lumbar-evoked potentials (LEPs) and motor-evoked potentials (MEPs) were measured during 20 and 60% of maximum voluntary contraction (MVC). Ten stimulations, randomly delivered, targeted 25% of maximum compound action potential for LEPs and 120, 140, and 160% of active motor threshold (aMT) for MEPs. The 7-week whole-body resistance training intervention included five exercises, e.g., knee extension (5 sets) and leg press (3 sets), performed twice weekly and was followed by 4 weeks of detraining.

**Results:**

Young had higher MVC (~ 63 N·m, *p* = 0.006), 1-RM (~ 50 kg, *p* = 0.002), and lower aMT (~ 9%, *p* = 0.030) than older adults at baseline. Young increased 1-RM (+ 18 kg, *p* < 0.001), skeletal muscle mass (SMM) (+ 0.9 kg, *p* = 0.009), and LEP amplitude (+ 0.174, *p* < 0.001) during 20% MVC. Older adults increased MVC (+ 13 N·m, *p* = 0.014), however, they experienced decreased LEP amplitude (− 0.241, *p* < 0.001) during 20% MVC and MEP amplitude reductions at 120% (− 0.157, *p* = 0.034), 140% (− 0.196, *p* = 0.026), and 160% (− 0.210, *p* = 0.006) aMT during 60% MVC trials. After detraining, young and older adults decreased 1-RM, while young adults decreased SMM.

**Conclusion:**

Higher aMT and MEP amplitude in older adults were concomitant with lower baseline strength. Training increased strength in both groups, but divergent modifications in cortico-spinal activity occurred. Results suggest that the primary locus of adaptation occurs at the spinal level.

**Supplementary Information:**

The online version contains supplementary material available at 10.1007/s00421-024-05443-0.

## Introduction

Aging is a complex process causing functional declines at both the cortical (Baudry et al. 2015; Clark and Taylor 2011) and spinal levels (Baudry et al. 2015; Geertsen et al. 2017; Hortobágyi et al. 2018; Kido et al. 2004). Neuronal atrophy, particularly within the motor cortex, can affect axonal regeneration potentially reducing motor cortex excitability (Fathi et al. 2010; Oliviero et al. 2006) and decreasing cortical inhibition (Christie and Kamen, 2014; Oliviero et al. 2006). Spinal motor-neurons, the last executors of neural commands from the cortex, are also susceptible to age-related changes such as a decline in population (Cruz-Sánchez et al. 1998; Tomlinson and Irving, 1977) and synaptic input reorganization. These changes can lead to a decrease in maximal force production, power, and physical function (Clark and Taylor 2011; Hunter et al. 2016).

Cortico-spinal excitability is evaluated using transcranial magnetic stimulation (TMS) to induce action potentials, producing a motor-evoked potential (MEP) (Barker and Jalinous 1985). Changes in MEP indicate the cortico-spinal tract’s integrity (Day et al. 1989; Kobayashi and Pascual-Leone 2003). During voluntary contraction, TMS causes a pause in electromyography (EMG), known as the cortical silent period (cSP) (Mills 1988). The duration of the cSP provides insights into intracortical inhibition (Inghilleri et al. 1993; Taylor et al. 1996), which varies depending on the target muscle (Yacyshyn et al. 2016; Gomez-Guerrero et al. 2023a) and contraction intensity (Gomez-Guerrero et al. 2023a).

Cortical and spinal excitability are inseparable from MEP responses (Taylor 2006), thus, electrical stimulation at the spinal level is needed for specific insight into spinal motor-neurons. Given the importance of lower-limb function for ambulation (Landin et al. 2016), which predicts disability and mortality (Guralnik et al. 1995; Millington et al. 1992), methodologies targeting lower-limb muscles in aging individuals are needed. Traditional peripheral-nerve stimulation has been questioned (McNeil et al. 2013), and direct spinal-cord stimulation at corticomedullary and thoracic levels can cause discomfort. In contrast, lumbar stimulation (LS), validated in healthy young adults (Škarabot et al. 2019a), has shown reliability during 20 and 60% muscular voluntary contraction (MVC) in active healthy adults (18–75 years old) (Gomez-Guerrero et al. 2023b) and is well-tolerated by young males (Brownstein et al. 2020), inducing an action potential in spinal motor-neurons and eliciting a lumbar-evoked potential (LEP) in the anterior thigh muscles’ EMG.

Strength training interventions are a safe and robust method to decelerate the aging process by enhancing functional capacity in untrained older adults (Siddique et al. 2022). In healthy young adults, strength training induces neural adaptations, during the first 3–4 weeks, by inducing plastic changes at the cortical (Weier et al. 2012; Goodwill et al. 2012) and spinal level (Aagaard et al. 2002; Holtermann et al. 2007). Specifically, increased MEP amplitude at 110–140% aMT was observed in m.rectus femoris (RF) within a recruitment curve (90–140% active motor threshold (aMT)) following twelve sessions of heavy-squat training (4 sets, 6–8 repetitions, at 80% 1-repetition maximum (1-RM)) in healthy young adults (Weier et al. 2012). A meta-analysis (Kidgell et al. 2017) reported that strength training may induce cortico-spinal adaptations in young adults, indicated by both increased MEP amplitude and decreased cSP duration. On the other hand, 6 sessions of strength training over 3 weeks in the ankle dorsiflexors did not change MEP amplitude but decreased cSP length in both untrained young and older adults (Christie and Kamen 2014); currently the only study to use TMS to evaluate a strength-training intervention in older adults.

Similarly, 2 to 3 weeks (6–9 training sessions in total) of strength training in older adults did not show spinal adaptations as measured by Hoffman-reflex (H-reflex) amplitude (Christie and Kamen 2014; Unhjem et al. 2020). Nevertheless, spinal adaptations have been documented following 3 to 14 weeks of strength training when measured during maximal (100% of MVC) (Aagaard et al. 2002) or submaximal (20 and 60% of MVC (Holtermann et al. 2007); and 10% of MVC (Vila-Chã et al. 2012)) contractions in young adults. Therefore, it is not presently clear whether adaptations in inhibitory pathways observed in young adults is due to an age effect per se or whether older adults may simply require more than 3 weeks/nine training session to achieve the same level of adaptation as the young. Consequently, it remains unclear whether neural plasticity resulting from strength training occurs at a cortical or spinal level or perhaps involves both (Siddique et al. 2022), and whether certain neural adaptations are specific to young and older age.

Finally, short- and long-term withdrawal (i.e., detraining) from strength training leads to decreased strength in young and older adults (Häkkinen et al. 2000, 1981). Although there is currently no specific investigation as to how cortical and spinal mechanisms affect the decrease in strength in young and older adults after a detraining period (Hortobágyi et al. 2021), some studies have reported a decrease in EMG activity and strength after a short-term (i.e., 3 to 6 weeks) detraining period in older adults (Häkkinen et al. 2000; Toraman 2005). Consequently, utilizing a training–detraining model would enhance confidence in interpreting causality from accompanying MEP and LEP changes along with strength level**.** Therefore, the aim of this study was to evaluate cortical and spinal adaptations in RF during a 7-week strength training period that included a 4-week detraining period in both young and older adults.

## Material and methods

### Participants

**Fig. 1:**
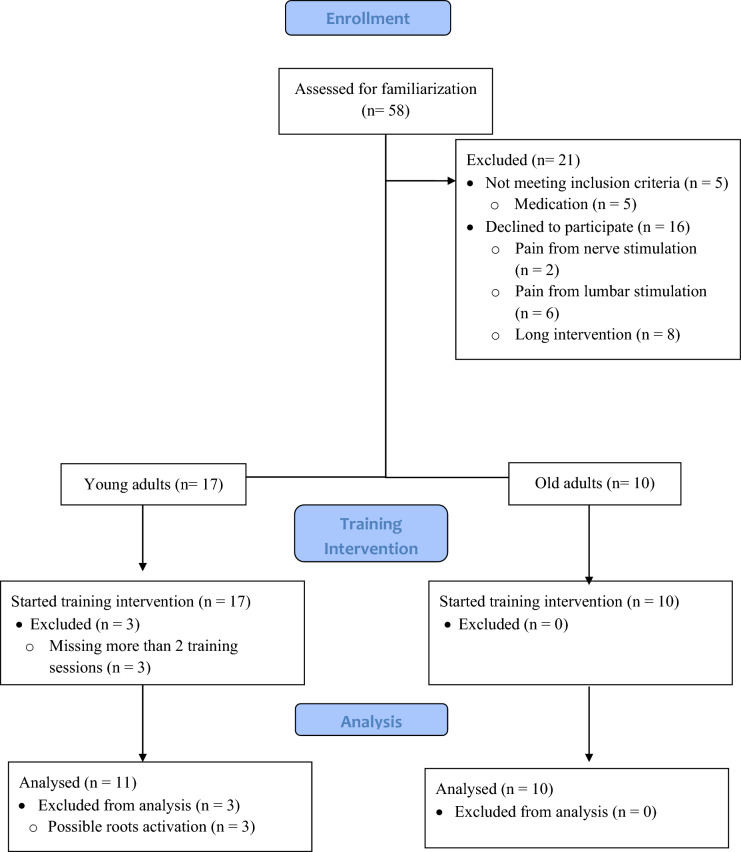
Flow chart of study enrollment, strength-training intervention, and analysis

Twenty-seven participants volunteered for the study (14 female). The recruitment process and exclusion of participants is shown in Fig. 1. Therefore, the data presented in Table 1 are representative of the 21 (11 young adults (6 female) and 10 older adults (6 female)) volunteers fulfilling all study requirements. All included participants were free from musculoskeletal injury in the lower-limbs for the last 6 months and neurologic illness, were not taking any medications known to affect the nervous system and had no contraindications to TMS, which was assessed via a health questionnaire (Rossi et al. 2011). Before testing, all participants were fully informed of the procedures and possible risks, and each participant provided written informed consent. The Ethical Committee of the University of Jyväskylä provided a statement for the study (857/13.00.04.00/2021) and the study was conducted in accordance with the ethical standards establish in the *Declaration of Helsinki* (2013).

**Table 1:** Mean ± standard deviation and statistical comparison of older versus younger adults of measurements during control

	Young adults	Older adults	Between-group *p*-value	95% CI [lower-bound, upper-bound]	Hedges’ *g*
Age (years)	27 ± 5	71 ± 4	–	–	–
Height (m)	1.74 ± 0.10	1.66 ± 0.06	–	–	–
Body mass (kg)	83.99 ± 24.23	74.73 ± 9.49	*p* = 0.272	[− 26.41, 7.89]	− 0.47
Body Mass Index (kg/m^2^)	26.78 ± 5.83	27.19 ± 3.30	*p* = 0.857	[− 4.58, 3.85]	− 0.08
Skeletal muscle mass (kg)	32.54 ± 7.88	27.59 ± 3.82	*p* = 0.088	[− 11.15, 0.66]	− 0.75
Body fat mass (kg)	26.14 ± 13.13	24.34 ± 9.12	*p* = 0.723	[− 12.23, 8.64]	− 0.15
MVC (N⋅m)	202 ± 53	139 ± 38	*p* = 0.006	[− 105.54, − 20.84]	− 1.31
1-RM (kg)	127 ± 42	77 ± 16	*p* = 0.002	[− 79.93, − 21.12]	− 1.51
M-max (mV)	2.65 ± 1.25	1.23 ± 0.50	*p* = 0.003	[− 2.32, − 0.53]	− 1.41
LEP stimulation intensity (mA)	262 ± 93	200 ± 77	*p* = 0.136	[− 144.89, 21.29]	− 0.69
aMT (%)	31 ± 6	40 ± 11	*p* = 0.030	[0.91, 16.33]	0.98

*CI* confidence interval, *m* meter, *kg* kilogram, *MVC* maximal voluntary contraction, *N* Newton, *1-RM* one-repetition maximum, *M-max* maximal compound action potential, *mV* millivolt, *LEP* Lumbar-evoked potential, *mA* milliampere, *aMT* active motor threshold

### Experimental set‑up

**Fig. 2:**
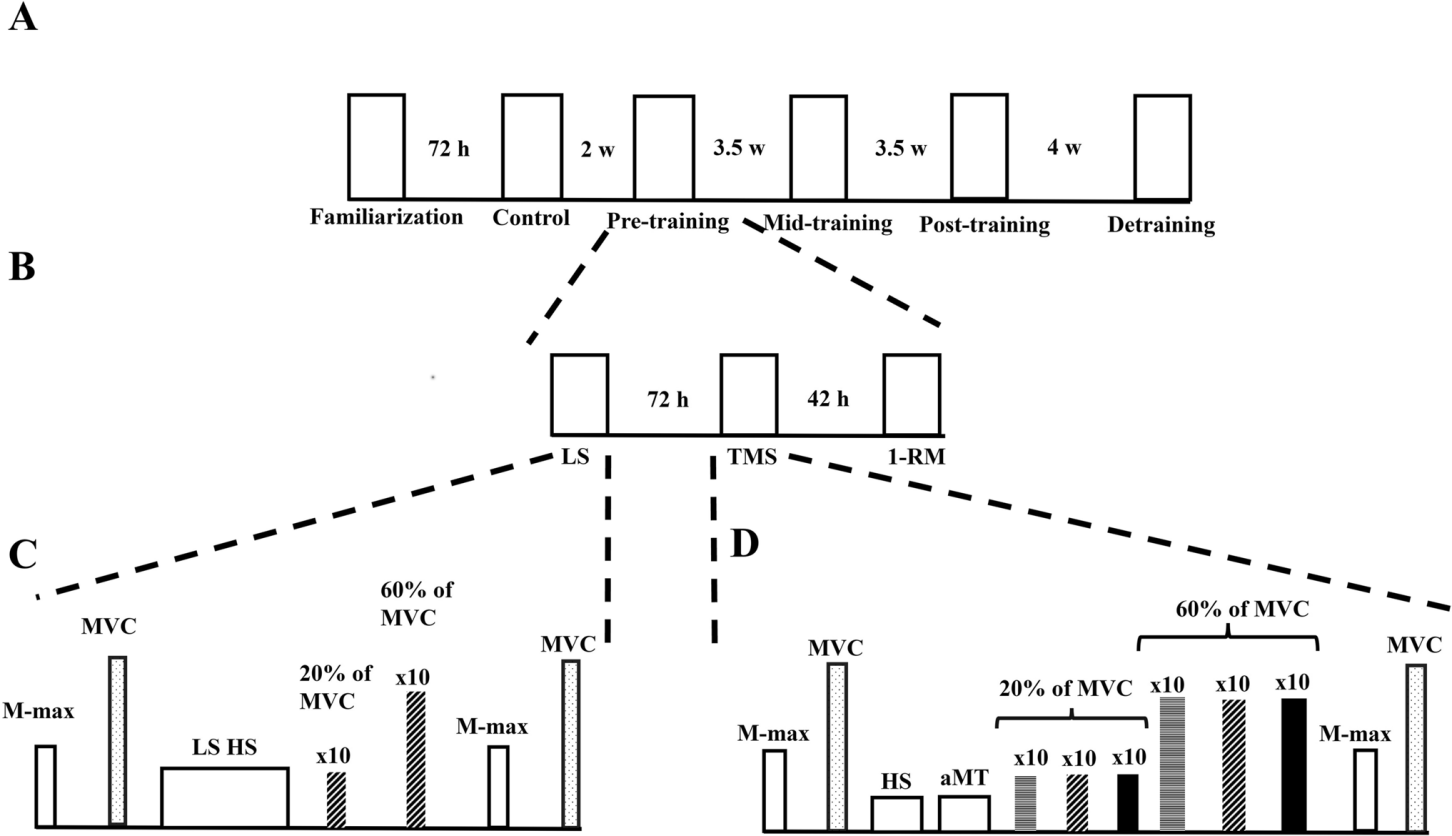
Description of the experimental timeline. **A** order of the six different testing periods. The time between testing sessions refers to the total time between one test period to the next. **B** Example of the testing sessions set up within a testing period. The time in-between the sessions is the minimum amount of time between test. **C** lumbar stimulation session set-up. **D** TMS stimulation sessions set-up. *H* hours, *W* weeks, *LS* lumbar stimulation, *TMS* transcranial magnetic stimulation, *1-RM* one-repetition maximum, *M-max* maximum compound action potential, *MVC* maximal voluntary contraction, *HS* hotspot, *aMT* active motor threshold

Participants visited the laboratory on five different testing periods and one familiarization session (Fig. 2A). All participants were instructed to maintain their regular dietary habits up to two hours prior to the testing session, consume 500 ml of water immediately before the test, abstain from consuming caffeine within the 12 h leading up to the examination, and refrain from engaging in strenuous physical activities 48 h preceding each testing session. The study’s initial phase consisted of a familiarization session in which participants were introduced to all instructions and stimulation parameters pertinent to the subsequent testing sessions. This session also served as a preliminary assessment of the LS placement and the determination of TMS intensity for active motor threshold (aMT). Then, testing periods were defined as control testing (Con), pre-training testing (Pre), mid-training testing (Mid), post-training testing (Post) and detraining testing (De) (Fig. 2A). Every testing period was structured the same: A LS session, a TMS session and a one-maximum repetition session (1-RM) conducted within a 7-day period. Sessions for each participant were consistently scheduled at the same time of the day, and there was a 48- to 72-h interval between LS, TMS and 1-RM (Fig. 2B).

To assess responses in RF, participants sat in a custom-built chair with a calibrated load cell (Faculty of Sport and Health Sciences, University of Jyväskylä, Finland) with the hip and knee at 90° flexion and the shank strapped by a non-elastic restraint ~ 2 cm superior to the ankle malleoli. The voltage signal originating from the load cell was calibrated and converted into torque (N·m). All measures were performed on the right (i.e., dominant) leg assessed by self-report of which foot they primarily kick a ball (van Melick et al. 2017).

Every session followed the same structure. Once the participant was secured to the dynamometer, the maximum compound action potential (M-max) was assessed in a relaxed condition (i.e., M-maxpre). As a warm-up, two contractions at ~ 50 and ~ 80% of estimated MVC were performed. Then, two MVC trials were performed 60 s apart (i.e., MVCpre). Verbal encouragement and visual feedback were provided to motivate participants to produce maximal effort and torque was recorded. The reliability of this method was excellent (CV = 4.6%; ICC = 0.987).

In every testing session, visual feedback was provided to the participants to produce the required submaximal torque and then a single LS or TMS stimulus was delivered manually. Contractions at 20 and 60% of MVC were held for 5–8 s, because RF MEP amplitude seemingly increases until 50–75% of MVC (Martin et al. 2006; Oya et al. 2008; Goodall et al. 2009; Škarabot et al. 2019a). Sets of ten stimulations were given per condition and per contraction level as a single block, giving a total of 40 LS and 60 TMS stimulations. To avoid fatigue, 30 s and 45 s rest was given between contractions during 20 and 60% of MVC, respectively, and 60 s and 180 s rest was given between the sets of 10 contractions. At the end of the protocol, M-max (M-maxpost) and MVC (MVCpost) were re-assessed (Fig. 2 C and D).

### Bipolar surface electromyography and torque

Muscle activity was recorded using adhesive Ag/AgCl electrodes (30 × 20 mm, BlueSensor N, Ambu, Penang, Malaysia) from RF according to SENIAM guidelines (Hermens et al. 2000). Skin was shaved, abraded with sandpaper, and wiped with alcohol before positioning the electrodes in a bipolar arrangement with a 20 mm center-to-center distance. Impedance was set < 2 kΩ, and the reference electrode was positioned on the patella. EMG electrode positions were marked with a permanent marker over the skin, photographs were taken and the distance from the iliac crest to the middle of the electrode pair was recorded. In addition, during the training period, the marks were redrawn by the research assistant after every training session. EMG data were sampled online at 3000 Hz, amplified (1000 ×) and bandpass filtered (16–1000 Hz; Neurolog System, Digitimer Ltd, UK) using CED Power1401-3 (Cambridge Electronic Design Ltd, Cambridge, UK).

Torque was sampled at 1000 Hz, amplified by a custom-built amplifier (ForAmps 1 v1.2, University of Jyväskylä, Finland) and converted by a 16-bit A/D board (CED Power1401-3, Cambridge Electronics Design, Cambridge, UK) in combination with Spike2 software (version 6.10, Cambridge Electronic Design, Cambridge, UK).

### Peripheral nerve stimulation

Transcutaneous electrical stimulation of the femoral nerve (32 mm cathode/anode arrangement; Polar Neurostimulation Electrodes, Espoo, Finland) was performed to elicit M-max in RF (1 ms squared pulse duration; Digitimer DS7AH, Hertfordshire, UK). Electrodes were placed 2 cm apart and positioned at each side of the femoral nerve, located by palpation and identification of the femoral artery (Walker et al. 2016). M-max was elicited by gradually increasing stimulator output intensity until the EMG response plateaued. To ensure a supramaximal response was elicited, this intensity was further increased by 50% and two individual simulations were given (Table 1).

### Lumbar stimulation

Transcutaneous electrical LS was used to elicit LEPs with a constant-current stimulator (1 ms square pulse duration; Digitimer DS7AH, Hertfordshire, UK) via self-adhesive electrodes (Polar Neurostimulation Electrodes, Espoo, Finland). Originally, the cathode (5 × 9 cm) was centered over the first lumbar vertebra and the anode (circular shape; 3.2 cm diameter) was placed on the midline of the vertebral column ~ 5 cm above the top edge of the cathode as described by Škarabot et al. (2019a).

Potential activation of ventral roots was examined from the onset latency of the LEP of an increasing stimulator intensity (Petersen et al. 2002) up to 25% of the M-max and also tracking LEP amplitude during increasing voluntary contraction while maintaining stimulator output intensity to that which produced a LEP amplitude of 25% of the M-max (Taylor et al. 2002). Should the ventral roots be activated by the stimulation procedures, onset latency would have shortened with an increase in stimulator intensity and LEP amplitude would have been the same during increased voluntary contraction (Petersen et al. 2002; Taylor et al. 2002). Three participants demonstrated no change in LEP amplitude with an increase in voluntary torque during offline analyses, and they were, therefore, removed from further analyses.

Dorsal root activation was assessed via paired LS with a 50 ms time delay, where the second LEP amplitude was compared to the first. Paired stimulation was conducted at rest, with the stimulator output intensity set to produce a LEP equivalent to 25% of the M-max. Evidence of dorsal-root activation would manifest as a decrease in the second LEP compared to the first, attributed to post-activation depression at the motor-neuron pool between the two stimuli (Hofstoetter et al. 2018). If the participant failed any of the tests (i.e., dorsal or ventral stimulation protocols), the electrodes were relocated 1 cm higher, until the participant passed all tests, or the anode was place between the third and fourth thoracic vertebrae. To ensure the placement was the same in all sessions, the distance from the 7th cervical vertebra to the anode (21.7 ± 4.1 cm) and from the bottom of the anode to the top of the cathode (3.7 ± 1.1 cm) (i.e., inter-electrode distance) were taken. All remaining participants showed no sign of the responses described and reported that they found LS to be tolerable. Once the placement was confirmed, stimulator intensity was kept to that which produced a LEP of 25% of the M-max at rest, and this stimulation intensity was used throughout the session (Table 1, Fig. 2C). The reliability of this method is reported in Gomez-Guerrero et al. (2023b) and considered moderate-to-good (ICC: 20% of MVC = 0.632; 60% of MVC = 0.520).

### Transcranial magnetic stimulation

Single TMS pulses were delivered using a MagStim 200^2^ magnetic stimulator (MagStim Co., Ltd., Whitland, UK) connected to a concave double-cone coil positioned over the left cortical hemisphere for RF with a posterior-to-anterior current orientation. The hotspot was defined at rest as the position eliciting the largest visible MEP recorded by EMG using the same intensity (approx. 50–70% stimulator output). Once the hotspot was found, the coil position was marked with a permanent marker on the scalp to maintain the same position throughout the protocol. Active motor threshold (aMT) was determined by increasing stimulator intensity in 5% steps, starting at 30% of the stimulator output. Thereafter, stimulator intensity was decreased in steps of 1% until clear MEPs (> 100 μV) were elicited in three out of five stimulations during unilateral isometric contractions of the right limb at 10% of MVC. Sets of ten single TMS stimulations were delivered in a random order for each of the assigned conditions (i.e., 120, 140 and 160% aMT) during unilateral isometric contractions at 20% and 60% of MVC (Fig. 2D). The reliability of these methods is reported in Gomez-Guerrero et al. (2023b) and considered good-to-excellent (ICC: 20% of MVC = 0.821–0.861; 60% of MVC = 0.901–0.941).

### Knee extension one-repetition maximum

All participants performed a bilateral concentric knee extension (David 200, David Health Solutions Ltd, Helsinki, Finland) one-repetition maximum (1-RM) test during the 5 test periods (Fig. 2A). First, each participant went through anthropometric analysis (Inbody 770, Inbody Co. Ltd, Seoul, Korea). Then, a 5 min cycling (1 kg load at 70 rpm) warm-up was performed followed by a series of submaximal warm-up sets (6 repetitions at an estimated 10-RM load, 3 repetitions at an estimated 6-RM load, 1-repetition at an estimated 3-RM load). Thereafter, single repetitions were performed until the participant could no longer lift the load from the beginning knee angle of ~ 85° to the required knee angle (≥ 170° knee angle), by visual inspection. The last successfully lifted load was recorded as the participant’s 1-RM and used to prescribe the load for the first and 4th week of training. Four-to-eight attempts where needed to calculate 1-RM with 1.25 kg precision. Verbal encouragement was provided to motivate participants to produce a maximal effort. 3 minutes rest were provided between attempts. The reliability of this method was excellent (CV = 8.4%; ICC = 0.991).

### Strength training sessions

Over the course of the 7 weeks of strength training, participants engaged in a total of 13 supervised sessions of conventional strength training. Mid-training testing was conducted after seven training sessions. Training sessions were conducted twice-a-week, with at least a 48-h break between sessions. The strength-training program was created following the guidelines provided by Fragala et al. (2019). The training program may be considered whole-body, targeting both upper- and lower-limbs, although we acknowledge that there were no dedicated abdominal or lower back exercises. Nevertheless, one or two exercises per muscle group were performed with a total volume of eight sets per muscle group for the lower-limbs and back/biceps and three sets for chest/triceps (Fragala et al. 2019). Each training session consisted of five different exercises for the upper- and lower-limbs: leg press, knee extension, bicep curl, smith-machine bench press and chest-supported seated row, in that order during normal training sessions. During testing sessions (Pre, Mid, Post), the order was: knee extension, leg press, smith-machine bench press, bicep curl. This training program closely resembles the most potent program for older adults identified in a meta-analysis (Borde et al. 2015). During the last set of the last session of the week, participants performed the maximum number of repetitions for each exercise to adjust either the volume or intensity (according to the estimated %RM) for the following week, so they could perform at least 8 repetitions.

All training sessions started with a warm-up, which consisted of 5 min of cycling and dynamic mobility exercises. During the initial training session, knee extension 1-RM testing was conducted. Subsequently, a 3–5 RM test was performed for the remaining exercise to determine and prescribe the training load. The rest of the sessions consisted of five (knee extension and bicep curl) and three sets (leg press, smith-machine bench press and chest-supported seated row) of 8–10 repetitions at 75–80% of 1-RM. The participants were asked to perform a 2 s-controlled eccentric phase, with no isometric phase and fast concentric phase.

A 4-week detraining period followed the strength-training period. Participants were allowed to maintain their normal physical activity (i.e., cycling, walking, running) during the whole intervention, but strength training was terminated during the detraining period.

### Data and statistical analyses

Offline analyses were performed with Spike2 software (version 6.10, Cambridge Electronic Design, Cambridge, UK) to manually obtain M-max amplitude and MVC torque. The other outcome measures were analyzed by a customized MATLAB script (version R2020b, The MathWorks, Inc., Natick, USA). Peak-to-peak amplitude of LEPs and MEPs were analyzed automatically between latencies-of-interest following LS or TMS, respectively. SP duration was defined, as the time from the stimulator artifact to the return of voluntary EMG (Damron et al. 2008). Torque was averaged over the 100 ms before the stimulator artifact (Škarabot et al. 2019b). LEP and MEP amplitude is represented as relative to M-max.

SPSS software (version 26.0, SPSS Inc., Chicago, USA) was used for all statistical methods. Means and standard deviation (SD) were calculated and reported throughout. Normality of the data was tested with the Shapiro–Wilk test and confirmed by a z-score with an acceptance of + 2 to − 2 (e.g., skewness score/skewness score_SE_ and kurtosis score/kurtosis score_SE_) and Q-plots for visualization. Data that did not fulfill those requirements were Log_10_ transformed, which then fulfilled the requirements for normality. A two-way repeated measures ANOVA (5 Time × 2 Group) was employed to assess most outcome variables (MVC, 1-RM, skeletal muscle mass, M-max, aMT, and silent periods of LEPs at 25% of the M-max and MEPs at 120, 140, 160% aMT) during contractions at 20 and 60% of MVC. When assumptions of sphericity were violated, Greenhouse–Geisser corrections were used. Post-hoc Bonferroni adjustments were used when significant main effects were found. To investigate the influence of strength training on the TMS- and LS-induced MEP/LEP amplitude, and to accommodate for missing data points and baseline variability, we employed a Linear Mixed Model (LMM) (Wilkinson et al. 2023). This model served as a robust framework for analyzing our data considering both fixed and random effects simultaneously. Cortico-spinal (MEPs at 120, 140, 160% aMT) and spinal (LEPs at 25% of the M-max) excitability at 20 and 60% of MVC were assessed using the LMM. The model included time (Con, Pre, Mid, Post, and De) and age group (young and older) as main effects and an interaction between age group (young and older) and time with participants as the random effect within the model. Bonferroni adjustments were used when significant main effects were found. Reliability, based on ICCs was categorized as poor (ICC < 0.5), moderate (ICC: > 0.5–< 0.75), good (ICC: > 0.75–< 0.9) and excellent (ICC: > 0.9) (Koo and Li 2016).Data are presented in the Tables by mean and SD, and in the results section by mean difference (MD), effect sizes are represented as partial eta-squared values (*η*_*p*_^*2*^ = small: 0.01, medium: 0.06, large: 0.14) for the factors of the ANOVA and post-hoc effect sizes reported as Hedge’s *g* (*g* = small: < 0.3, medium: 0.3–0.8, large: > 0.8). Αlpha was set at 0.05.

## Results

### Baseline between-group comparisons

Main effects for Group were observed for 1-RM (F _(1,19)_ = 15.94, *p* = 0.001, *η*_*p*_^*2*^ = 0.46), MVC (F _(1,19)_ = 9.60, *p* = 0.006, *η*_*p*_^*2*^ = 0.34), M-max (F _(1,19)_ = 20.86, *p* < 0.001, *η*_*p*_^*2*^ = 0.53), aMT (F _(1,19)_ = 11.75, *p* = 0.038, *η*_*p*_^*2*^ = 0.21), MEP amplitude during 60% of MVC with 120% aMT (F _(1,19)_ = 4.65 *p* = 0.044) and 140% aMT (F _(1,19)_ = 4.62 *p* = 0.045), MEP silent period during 20% of MVC with 120% aMT (F _(1,19)_ = 13.96, *p* = 0.001, *η*_*p*_^*2*^ = 0.42), LEP silent period duration during 20% of MVC (F _(1,19)_ = 5.60, *p* = 0.029, *η*_*p*_^*2*^ = 0.229), MEP silent period during 60% of MVC with 120% aMT (F _(1,19)_ = 23.39, *p* < 0.001, *η*_*p*_^*2*^ = 0.650), and LEP silent period duration during 60% of MVC (F _(1,19)_ = 23.39, *p* < 0.001, *η*_*p*_^*2*^ = 0.552).

**Fig. 3:**
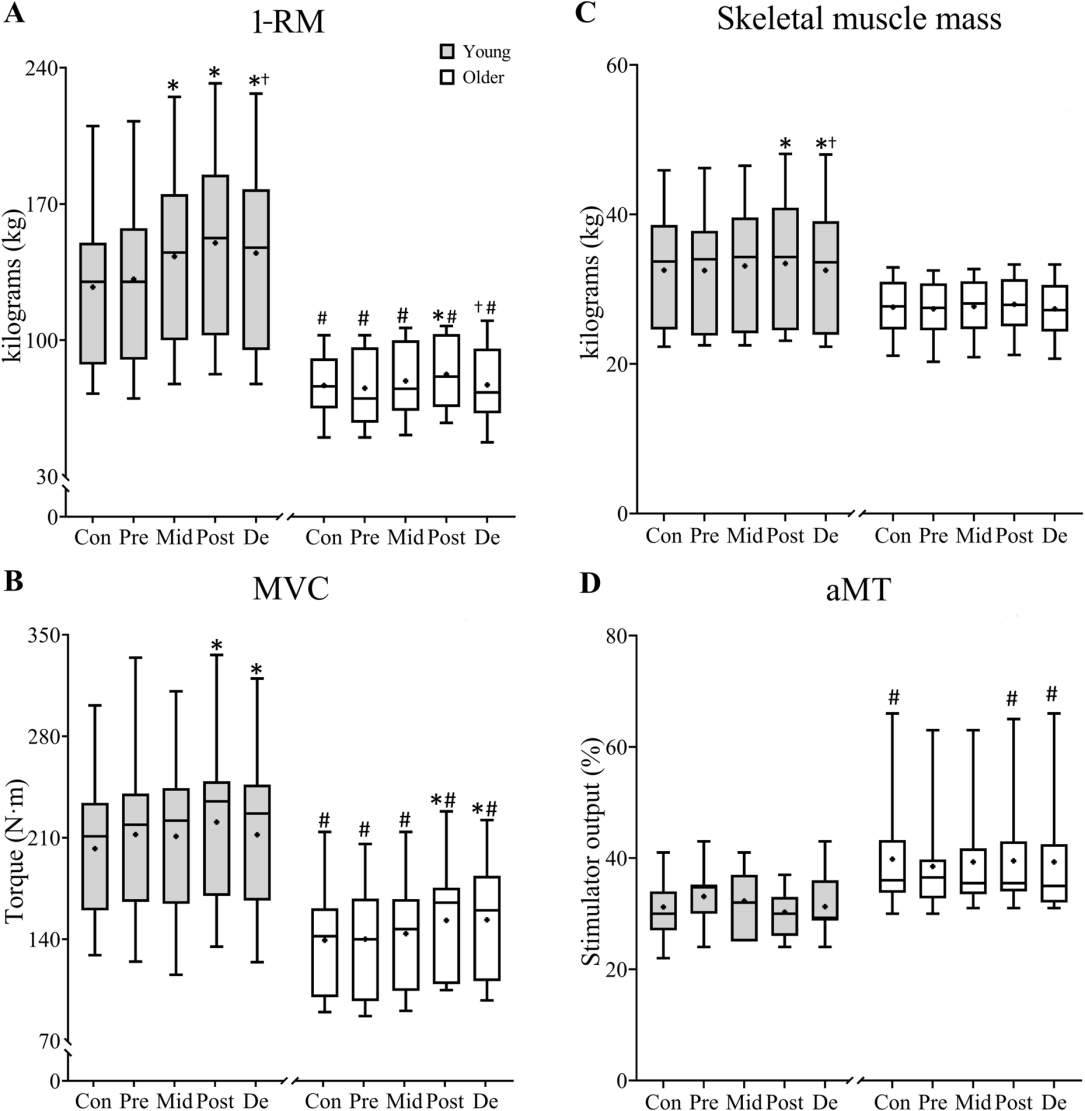
Box and whiskers plots showing the comparisons of group and time effect in young and older adults for **A** 1-RM, **B** MVC, **C** skeletal muscle mass and **D** aMT. Each figure shows quartiles and whiskers (minimum and maximum), the median (line in the box), mean (+ in the box) for each group (young: filled box and older: blank box) and session. *p < 0.05 post hoc within-group analysis compared to pre-training. *p* < 0.05 post hoc within-group analysis compared to post-training. #*p* < 0.05 post hoc between-group analysis compared to the older group

**Fig. 4:**
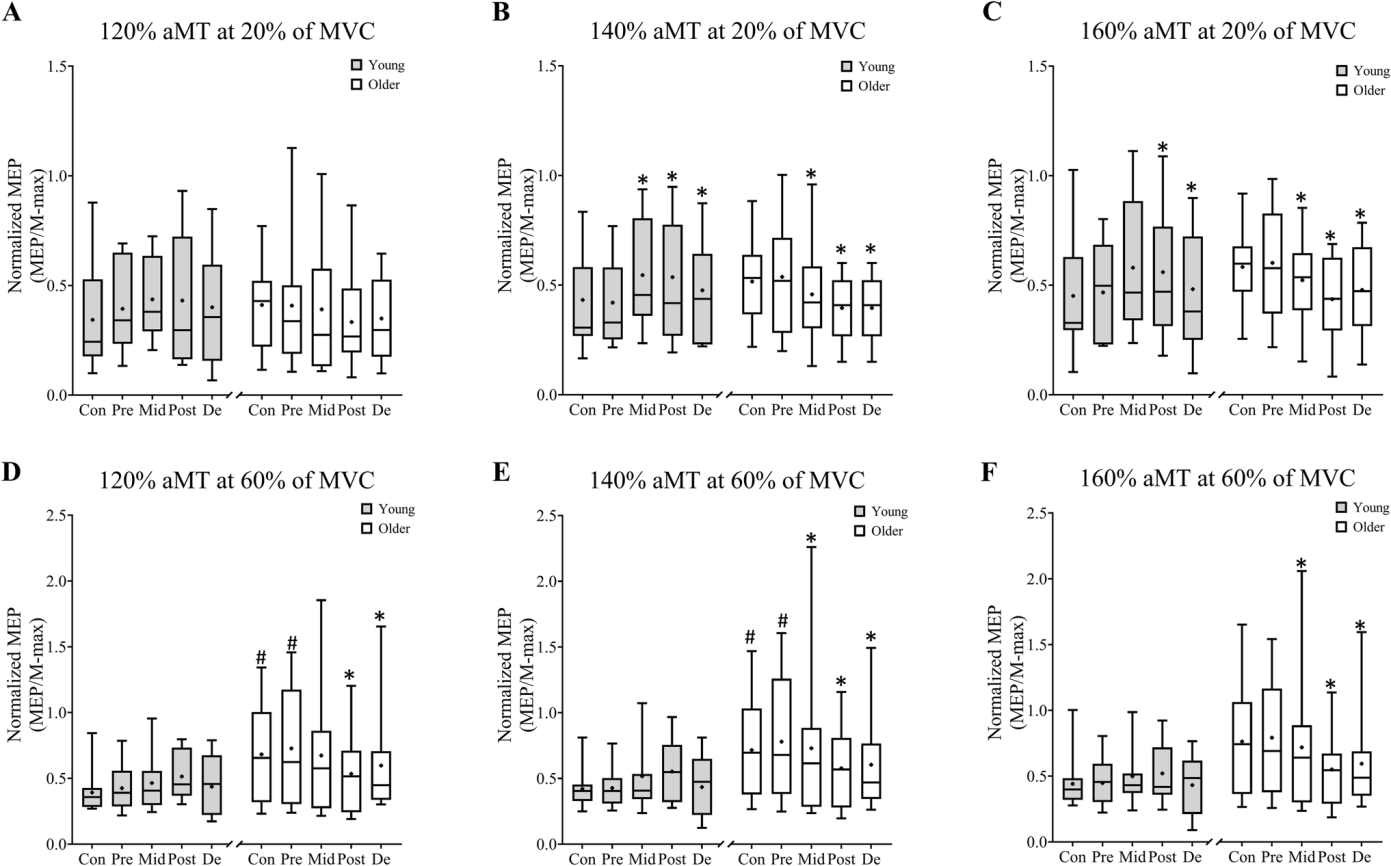
Box and whiskers plots showing the comparisons of group and time effect in young and older adults for different aMT intensities at 20% of MVC (120% aMT: **A**; 140% aMT: **B**; 160% aMT: **C** and 60% of MVC (120% aMT: **D**; 140% aMT: **E**; 160% aMT: **F**).Each figure shows quartiles and whiskers (minimum and maximum), the median (line in the box), mean (+ in the box) for each group (young: filled box and older: blank box) and session. *p < 0.05 post hoc within-group analysis compared to pre-training. #*p* < 0.05 post hoc between-group analysis compared to the older group

During the first measurement session (i.e., control), young adults were stronger than older adults, and had a higher M-max and lower aMT (Table 1 and Fig. 3). Further, during control, MEP amplitude at 120% and 140% aMT was greater in the older group during 60% of MVC (Fig. 4D and E). Silent period duration was longer in older adults during both 20% of MVC (99 ± 15 ms versus 117 ± 18 ms, p = 0.027) and 60% of MVC (94 ± 12 ms versus 121 ± 20 ms, *p* = 0.001) when stimulated at 120% aMT, and during 60% of MVC from LS (62 ± 7 ms versus 82 ± 20 ms, *p* = 0.006) (see supplementary material).

### Training-induced adaptations

For 1-RM, main effects for Time (F _(2.3,42.9)_ = 28.29, *p* < 0.001, *η*_*p*_^*2*^ = 0.60) and Time*Group interaction (F _(2.3,42.9)_ = 11.06, *p* < 0.001, *η*_*p*_^*2*^ = 0.38) were observed. Post-hoc comparisons showed that young adults increased from Pre to Post (*p* < 0.001) and then decreased from Post to De (*p* = 0.011, Fig. 3A). Older adults did not increase statistically Pre to Post but did Mid to Post (*p* = 0.027) and they also decreased Post to De (*p* = 0.012).

MVC demonstrated a significant main effect for Time (F _(4,76)_ = 10.13, *p* < 0.001, *η*_*p*_^*2*^ = 0.35). Post-hoc analysis showed that young adults increased significantly Mid to Post (*p* = 0.024) and older increased significantly Pre to Post (*p* = 0.014, Fig. 3B).

Skeletal muscle mass demonstrated a significant main effect for Time (F _(2.5,47.8)_ = 3.16, *p* < 0.001, *η*_*p*_^*2*^ = 0.323). Here, only young adults increased Pre to Post (*p* = 0.009) and then decreased Post to De (*p* < 0.001, Fig. 3C).

Significant main effects for Time and Time*Group interaction were observed for MEP amplitude during 20% of MVC at 120% aMT (Time: F _(4,1021)_ = 3.09, *p* = 0.015; Time*Group: F _(4,1021)_ = 4.10, *p* = 0.003), 140% aMT (Time: F _(4,1021)_ = 4.89, *p* = 0.001; Time*Group: F _(4,1021)_ = 14.44, *p* < 0.001), 160% aMT (Time: F _(4,1021)_ = 8.12, *p* < 0.001; Time*Group: F _(4,1021)_ = 4.10, *p* = 0.003). In the young adults, significant increases occurred Pre to Post with 140% aMT (*p* = 0.023) and Pre to Mid at 160% aMT (*p* = 0.005). In older adults, significant decreases were observed Pre to Post at 140% (*p* < 0.001) and 160% (*p* < 0.001) aMT (Figure, Fig. 4B and C).

Significant main effects for Time and Time*Group interaction were observed for MEP amplitude during 60% of MVC at 120% aMT (Time: F _(4,1021)_ = 4.24, *p* = 0.002; Time*Group: F _(4,1021)_ = 10.53, *p* < 0.001), 140% aMT (Time: F _(4,1021)_ = 7.97, *p* < 0.001; Time*Group: F _(4,1021)_ = 13.69, *p* < 0.001), 160% aMT (Time: F _(4,1021)_ = 13.50, *p* = 0.002; Time*Group: F _(4,1021)_ = 14.08, *p* < 0.001). Post-hoc comparisons showed that only older adults decreased Pre to Post with all stimulation intensities (*p* < 0.001, Fig. 4D–F).

Significant main effects for Time (F _(4,1021)_ = 3.09, *p* = 0.015) and Time*Group interaction (F _(4,1021)_ = 4.10, *p* = 0.003) were observed for LEP amplitude during 20% of MVC. Young adults significantly increased Pre to Post (*p* < 0.001) and subsequently decreased Post to De (*p* < 0.001). Also, in the young adults, there was a significant decrease from Con to Pre (*p* = 0.022). In older adults, a significant decrease occurred Pre to Post (*p* < 0.001) (Table 2).

**Table 2:** Mean ± standard deviation and statistical results from Linear Mix Models fixed effects of normalized LEP amplitude (LEP/M-max) for young and older groups at different contraction intensities and post-hoc comparison

	Control	Pre-training	Mid-training	Post-training	Detraining	Time *p*-value	Time*Group *p*-value	Group *p*-value
20% MVC								
Young adults	0.36 ± 0.13^*^	0.30 ± 0.10	0.31 ± 0.23	0.48 ± 0.23^*^	0.36 ± 0.22^+^	*p* = 0.003	*p* < 0.001	*p* = 0.857
95% CI	[0.30, 0.43]	[0.21, 0.39]	[0.26, 0.51]	[0.37, 0.59]	[0.30, 0.48]			
Older adults	0.35 ± 0.11	0.40 ± 0.21	0.37 ± 0.22	0.30 ± 0.10^*^	0.35 ± 0.13			
95% CI	[0.30, 0.43]	[0.30, 0.49]	[0.25, 0.51]	[0.18, 0.42]	[0.25, 0.45]			
60% MVC								
Young adults	0.51 ± 0.22^*^	0.41 ± 0.21	0.34 ± 0.21^*^	0.47 ± 0.20	0.41 ± 0.20	*p* < 0.001	*p* < 0.001	*p* = 0.313
[95% CI]	[0.39, 0.62]	[0.29, 0.54]	[0.25, 0.54]	[0.33, 0.60]	[0.31, 0.55]			
Older adults	0.50 ± 0.21	0.51 ± 0.23	0.48 ± 0.30	0.48 ± 0.25	0.51 ± 0.24			
[95% CI]	[0.39, 0.63]	[0.37, 0.63]	[0.34, 0.64]	[0.34, 0.62]	[0.39, 0.64]			

*MVC* maximal voluntary contraction, *LEP* lumbar-evoked potential, *M*-*max* maximal compound action potential, *CI* confidence intervals

*p < 0.05 post hoc within-group analysis compared to pre-training

*p* < 0.05 post hoc within-group analysis compared to post-training

Significant main effects for Time (F _(4, 1021)_ = 8.45, *p* < 0.001) and Time*Group interaction (F _(4, 1021)_ = 6.66, *p* < 0.001) were LEP amplitude during 60% of MVC. Post hoc showed that young significantly decreased Con to Pre (*p* < 0.001), further decreased Pre to Mid (*p* = 0.023), and then increased Mid to Post (*p* < 0.001) (Table 2).

## Discussion

This study addressed the lack of knowledge regarding cortico-spinal and spinal adaptations to short-term strength training and detraining in young and older adults, specifically in the lower-limbs. The results showed an increase in maximum strength for both groups after seven weeks of training and a partial reversal following four weeks of detraining. The main result of interest was that young adults demonstrated increased cortico-spinal and spinal excitability as a consequence of training, but older adults showed the opposite, i.e., decreased cortico-spinal and spinal excitability. Furthermore, the present study revealed that older adults required greater stimulation intensity to elicit an MEP (i.e., aMT), cortico-spinal excitability at higher contraction intensity was greater, and cortical and spinal inhibition was greater in older adults at baseline accompanying the between-group strength differences suggesting an effect of age.

The observed differences in 1-RM and MVC between young and older adults would be expected due to the age-related reduction in maximal strength (Bemben et al. 1991). Further, both young and older adults responded positively to a short-term strength training intervention observed through increases in 1-RM and MVC, again as expected from previous studies (Christie and Kamen 2014; Häkkinen et al. 2000; Walker and Häkkinen 2014). The 1-RM increases in the present study of Δ14% and Δ9% in young and older adults, respectively, are similar to those reported by Walker and Häkkinen (2014) over ten weeks of training. Interestingly, increases in lean leg mass in that study occurred only in the younger group (Walker et al. 2014), and only the young group increased skeletal muscles mass in the present study. These converging results suggest that neural mechanisms, rather than morphologic, may be responsible for increased maximal strength in previously untrained older adults when initiating strength training. Previously untrained young adults, on the other hand, appear to improve maximal strength through a combination of neural and morphologic mechanisms.

### Cortico-spinal excitability

An interesting observation was the consistent decrease in MEP excitability in the older group, independent of the contraction intensity. These changes became apparent as early as three weeks into the training. Our results differ from those reported by Christie and Kamen (2014) who reported that two weeks of training (six training sessions) did not induce significant changes in MEP amplitude in the m.tibialis anterior. The authors noted decreases of 4–6% (n.s) in MEP amplitude in the older adults. The magnitude of those results was similar to our results (− 7 to 8%) after 3 weeks/6 sessions of strength training but ours further decreased (to – 12 to 21%) after 7 weeks/ 13 sessions of strength training. Therefore, cortico-spinal adaptation in older adults seems to require more training duration than in young adults.

Furthermore, the interaction, and within-group changes of LEP amplitude parallel those of MEP amplitude; older adults showing a reduction in LEP amplitude at 20% of MVC. In addition, LEP amplitude increased in the young group from pre- to post-training at 20% of MVC and then decreased back to baseline after detraining. No clear or systematic changes were observed in either group during 60% of MVC trials, and the observed fluctuations may be due to the relatively high typical error/reliability values of this method (Gomez-Guerrero et al. 2023b). Nevertheless, one previous study investigating short-term strength training effects (Ansdell et al. 2020) observed no changes in MEP nor LEP amplitude at a group level; where large inter-individual differences apparent with approximately half of the group increasing and half decreasing amplitude after 12 sessions of 4 sets of 6–8 back squat repetitions. In contrast, Lundbye-Jensen et al. (2005) demonstrated decreased cortico-spinal excitability in untrained healthy young adults after thirteen training sessions spread over 4 weeks. This effect was observed at several higher TMS stimulator output intensities (160–220% rMT), similar to our differences observed at 140 and 160% aMT. The authors discussed that those changes could potentially be at subcortical levels through changes in spinal motor-neuron firing rate and/or intrinsic firing properties, although this was not specifically tested. In support, Vila-Chã et al. (2012) and Aagaard et al. (2002) observed spinal adaptations, through better modulation of inhibitory pathways, after 3 weeks and 14 weeks of strength training in younger adults. Thus, in the present study, the older group adapted to the training by reducing their MEP amplitude down to the level of the young and these adaptations could be at a spinal level.

Conversely, small magnitude but statistically significant increases in MEP excitability occurred in the young group after strength training, as has been previously reported (Goodwill et al. 2012; Kidgell et al. 2017; Weier et al. 2012). Goodwill et al. (2012) and Weier et al. (2012) found that a short-term training intervention, twelve sessions, produced an increase in MEP amplitude of RF when measured at 10% of MVC. Those results are in line with our results at 20% of MVC. However, and importantly for our interpretation, MEP excitability assessed at 60% of MVC did not show significant changes in the young. Strength training and maximal strength has been proposed as a specific skill (Buckner et al. 2017), and 12 sessions of arm flexion–extension visuomotor tracking skill training (Lundbye-Jensen et al. 2005) along with 12 sessions of 3 s concentric and 4 s eccentric tempo-controlled bicep curl strength training (Leung et al. 2017) has been shown to increase MEP amplitude after four weeks. Since the participants were required to hold the force level constant prior to stimulation (~ 2 s), it may be that lower force levels challenge the sensorimotor system to a greater extent than higher contraction levels, as previously evident in force steadiness tasks (Laidlaw et al. 2000). Therefore, we propose that the statistically significant but small magnitude changes in excitability in the young observed only during 20% of MVC trials reflect the sensorimotor integration needed for force steadiness, a so-called ‘skill element’ of strength training.

Our results showed higher aMT in older adults compared to younger adults, which is an indicator of cortico-spinal excitability (Pascual-Leone et al. 1995; Wassermann 2002). Should this reflect a decline in cortico-spinal excitability with age, as interpreted in previous studies (Bashir et al. 2014; Cirillo et al. 2011), this would directly conflict the MEP amplitude data of the present study. The aging process may lead to reduced activation of cortico-spinal neurons or disrupted synchronization among these neurons leading to a cancelation phase (Pitcher et al. 2003; Magistris et al. 1998). Notably, despite the impact of strength training and subsequent detraining on MEP and LEP amplitudes, aMT remained unchanged across interventions and age groups suggesting a discrepancy between the measures as an indicator of excitability. Previous studies have discussed (Wassermann 2002; Hassanlouei et al. 2017) that caution is advised in interpreting aMT due to factors such as a reduction in motor cortex size (Marner et al. 2003; Salat et al. 2004) and increase in skull thickness (Lillie et al. 2016) with age that potentially increases the coil-to-cortex distance, meaning a requirement for higher intensities for action potential generation. It may be that the between-group differences in aMT of the present study is due to cortex size or skull thickness rather than cortico-spinal excitability per se. While our study did not directly address these factors, our results underscore the need for further investigation to identify the precise mechanisms.

In addition, at higher contraction intensities in the present study, the older group showed greater MEP amplitude than the younger group at baseline. Further, Hassanlouei et al. (2017) showed that individuals engaged in higher physical activity (> 10,000 steps/day) demonstrated lower MEP amplitude in m.vastus lateralis than the ones with low physical activity (< 10,000 steps/day), independent of age. Moreover, cast immobilization has been shown to increase cortico-spinal excitability, when measured at 120% rMT (Roberts et al. 2007). Both studies discuss that modulation of different inhibitory pathways at the cortical level could modify cortico-spinal excitability due to the lack of exercise. These data suggest that better trained muscles for gross force production, remembering that older adults are generally less physically active than young (Martin et al. 2014), are characterized by lower cortico-spinal excitability responses to TMS.

### Cortical and spinal inhibition

Our results showed that neither strength training nor detraining affected MEP or LEP cSP duration. This is somewhat unexpected as meta-analyses have shown reductions in cSP duration following strength training (Kidgell et al. 2017; Mason et al. 2019), at least in young adults. Nevertheless, within these meta-analyses there have been studies showing no changes in cSP, thus, our data is not without precedent. For example, 12 strength training sessions of 4 sets of 6–8 repetitions with 80% 1-RM using 3 s concentric and 4 s eccentric tempo-controlled contractions led to no changes in biceps brachii cSP in healthy young adults (Kidgell et al. 2011).

At baseline, our results showed that MEP SP at 120% aMT and LEP SP were significantly longer for the older group independently of the contraction intensity used. cSP is an indication of intracortical inhibition (Inghilleri et al. 1993) mediated by Gamma-aminobutyric acid (GABA) inhibitors, particularly involving the activity of GABA_B_ receptors (Siebner et al. 1998). Consequently, prolonged cSP indicates greater GABA_B_ activity and longer intracortical inhibition in the older group. These results contradict previous findings, where SP durations were reported shorter (Christie and Kamen 2014; Sale and Semmler 2005) or not different (Fujiyama et al. 2012) comparing younger and older adults at baseline. However, it should be noted that MEPs were either of similar amplitude (Christie and Kamen 2014) or smaller (Sale and Semmler 2005) than the younger adults in those previous studies, which contrasts the higher MEP and LEP amplitudes for the older adults here. Given the correlation between cSP and MEP amplitude (Orth and Rothwell 2004), it is plausible that normalization of cSP to MEP amplitude in the older group might have led to an interpretation of increased inhibition in older adults, due to the decreased MEP size and no changes in cSP in the older adults.

Moreover, the present study showed decreased MEP amplitude following strength training while the SP duration from cortical and spinal stimulation remained unchanged. Therefore, normalizing the SP to MEP or LEP amplitude, would modify the interpretation of excitatory and inhibitory processes influencing the observed outcomes. Thus, the observed decrease in MEP/LEP amplitude and the conserved SP may indicate greater contribution of cortical and/or spinal inhibition in older adults after training, which may improve movement efficiency and result in increased strength.

### Strengths and limitations

This study is the first to provide evidence of cortical and spinal excitability and inhibition adaptations to a 7-week strength training intervention in young and older adults. In addition, it also provides information from a detraining period, which strengthens inferences that can be drawn from the causality of the intervention. Furthermore, cortico-spinal responses were recorded during different contraction intensities. Clearer between-group differences (at baseline) were observable at 60% of MVC compared to 20% of MVC, and this finding could direct future studies comparing differences between groups. In addition, the detraining period provides support that the intervention caused the observed alterations in the outcome measures and helps to identify the mechanisms of improved strength. The young increased and decreased both strength and muscle mass concomitantly, suggesting that morphologic adaptations were a large factor in the strength increase. Conversely, the older adults maintained both strength and the altered MEP/LEP amplitude after detraining suggesting that neural adaptations predominantly underpinned the strength gain.

As a limitation, the strength-training program was performed dynamically and mainly bilaterally. Thereby, the unilateral isometric test was non-specific and could have influenced the ability to identify neural adaptations. TMS paired-pulse paradigms (i.e., SICI, LICI, ICF), peripheral stimulation paradigms (H-reflex) and/or paired H-reflex -TMS (cortical recurrent inhibition) were not measured in this study because an increased number of contractions per session would have increased the risk of fatigue. This could have provided more specific information about how strength training modulates cortical and spinal inhibitory process in young and older adults alongside cortico-spinal and spinal excitability.

## Conclusions

The present study has shown maximal strength, cortico-spinal excitability and cortical and spinal differences between young and older group at baseline, that are believed to be related to the aging process. Furthermore, the short-term strength-training intervention showed improved strength in both groups and that early cortico-spinal adaptations might be age-dependent as well as specific to contraction level. The decrease in MEP amplitude at 60% of MVC indicates cortico-spinal adaptations in the older adults. In addition, LEP amplitude changes in young and older could suggest spinal adaptation as the primary site after strength training in young and older adults, proving strength training as a beneficial tool to decelerate aging.

### Supplementary Information

Below is the link to the electronic supplementary material.

Supplementary file1 (DOCX 19 KB)

Supplementary file2 (DOCX 18 KB)

## Data Availability

The datasets generated and/or analyzed during the current study are available from the corresponding author on reasonable request.

## Abbreviations

1-RM: One-maximum repetition
aMT: Active motor threshold
ANOVA: Analysis of variance
CI: Coefficients intervals
CV: Coefficient of variance
cSP: Cortical silent period
EMG: Electromyography
ES: Effect size
GABA: Gamma-aminobutyric acid
H-reflex: Hoffmann’s reflex
Hz: Hertz
ICC: Intra-class correlation
ICF: Intra-cortical facilitation
LEP: Lumbar-evoked potential
LICI: Long intracortical inhibition
LS: Lumbar stimulation
MEP: Motor-evoked potential
M-max: Maximum compound action potential
MVC: Maximal voluntary contraction
RF: Rectus femoris
SICI: Short intracortical inhibition
SMM: Skeletal muscle mass
TMS: Transcranial Magnetic Stimulation
