# Supplementary material for: Cortical and spinal responses to short-term strength training and detraining in young and older adults in rectus femoris muscle
Source: Eur J Appl Physiol. 2024 Mar 5;124(7):2209–23. doi: 10.1007/s00421-024-05443-0 (PMC11199260; doi:10.1007/s00421-024-05443-0)
Supplement: Supplementary file 1 — Supplementary file1 (DOCX 19 KB) [file 421_2024_5443_MOESM1_ESM.docx]

Supplementary Table 1. Mean ± standard deviation and two-way ANOVA pairwise comparisons of MEP and LEP SP for young and older adults at 20% of MVC with post-hoc comparisons.

|  | Control | Pre-training | Mid-training | Post-training | Detraining | Time  p-value | Time*Group  p-value | Group  p-value |
| --- | --- | --- | --- | --- | --- | --- | --- | --- |
| 20% of MVC |  |  |  |  |  |  |  |  |
| MEP SP (ms) |  |  |  |  |  |  |  |  |
| 120% aMT |  |  |  |  |  |  |  |  |
| Young adults | 99 ± 15 | 107 ± 19 | 99 ± 11 | 96 ± 11 | 102 ± 17 | p = 0.264 | p =0.847 | p = 0.001 |
| Older adults | 117 ± 18 # | 119 ± 15 | 115 ± 15# | 113 ± 14# | 115 ± 12 |  |  |  |
| 140% aMT |  |  |  |  |  |  |  |  |
| Young adults | 119 ±16 | 128 ± 19 | 123 ±15 | 119 ±14 | 125 ± 17 | p = 0.527 | p = 0.681 | p = 0.121 |
| Older adults | 138 ± 33 | 138 ± 33 | 135 ± 27 | 125 ±17 | 131 ± 15 |  |  |  |
| 160% aMT |  |  |  |  |  |  |  |  |
| Young adults | 139 ± 23 | 144 ± 16 | 141 ± 22 | 143 ± 21 | 139 ± 20 | p = 0.287 | p = 0.264 | p = 0.640 |
| Older adults | 149 ± 38 | 147 ± 38 | 136 ± 23 | 148 ± 31 | 151 ± 37 |  |  |  |
| LEP SP (ms) |  |  |  |  |  |  |  |  |
| Young adults | 77 ± 8 | 73 ± 7 | 69 ± 7 | 72 ± 10 | 71 ± 10 | p = 0.653 | p = 0.221 | p = 0.029 |
| Older adults | 81 ± 16 | 82 ± 11# | 90 ± 28# | 90 ± 33 | 83 ± 21 |  |  |  |

MVC = maximal voluntary contraction; MEP = motor-evoked potential; SP = silent period; ms = milliseconds; aMT = active motor threshold LEP = lumbar-evoked potential; M-max = maximal compound action potential

# = p < 0.05 post hoc between group analysis compared to the young group
