# Supplementary material for: Cortical and spinal responses to short-term strength training and detraining in young and older adults in rectus femoris muscle
Source: Eur J Appl Physiol. 2024 Mar 5;124(7):2209–23. doi: 10.1007/s00421-024-05443-0 (PMC11199260; doi:10.1007/s00421-024-05443-0)
Supplement: Supplementary file 2 — Supplementary file2 (DOCX 18 KB) [file 421_2024_5443_MOESM2_ESM.docx]

Supplementary Table 2. Mean ± standard deviation and two-way ANOVA pairwise comparisons of MEP and LEP SP for young and older adults at 60% of MVC with post-hoc comparisons.

|  | Control | Pre-training | Mid-training | Post-training | Detraining | Time  p-value | Time*Group  p-value | Group  p-value |
| --- | --- | --- | --- | --- | --- | --- | --- | --- |
| 60% of MVC |  |  |  |  |  |  |  |  |
| MEP SP (ms) |  |  |  |  |  |  |  |  |
| 120% aMT |  |  |  |  |  |  |  |  |
| Young adults | 94 ± 12 | 98 ±10 | 90 ± 11 | 91 ± 13 | 91 ± 17 | p = 0.153 | p = 0.610 | p < 0.001 |
| Older adults | 122 ± 20# | 121 ±13# | 120 ± 13# | 114 ± 16# | 112 ± 12# |  |  |  |
| 140% aMT |  |  |  |  |  |  |  |  |
| Young adults | 124 ± 31 | 127 ± 25 | 115 ±19 | 113 ± 21 | 120 ±37 | p = 0.572 | p = 0.233 | p = 0.381 |
| Older adults | 122 ± 10 | 123 ± 9 | 131 ± 14 | 124 ± 7 | 129 ± 8 |  |  |  |
| 160% aMT |  |  |  |  |  |  |  |  |
| Young adults | 143 ± 38 | 146 ± 35 | 142 ± 29 | 142 ± 25 | 143 ±28 | p = 0.721 | p = 0.898 | p = 0.769 |
| Older adults | 141 ± 35 | 145 ± 37 | 143 ± 23 | 136 ± 24 | 136 ± 20 |  |  |  |
| LEP SP (ms) |  |  |  |  |  |  |  |  |
| Young adults | 62 ± 7 | 63 ± 8 | 61 ± 7 | 56 ± 9 | 54 ± 7 | p = 0.036 | p = 0.076 | p < 0.001 |
| Older adults | 83 ± 20# | 72 ± 15 | 71 ± 13# | 69 ± 12# | 79 ± 12# |  |  |  |

MVC = maximal voluntary contraction; MEP = motor-evoked potential; SP = silent period; ms = milliseconds; aMT = active motor threshold LEP = lumbar-evoked potential; M-max = maximal compound action potential

# = p < 0.05 post hoc between group analysis compared to the older group
